# New Concepts on the Pathophysiology of Acute Coronary Syndrome

**DOI:** 10.31083/j.rcm2404112

**Published:** 2023-04-17

**Authors:** Deqiang Yuan, Jiapeng Chu, Jun Qian, Hao Lin, Guoqi Zhu, Fei Chen, Xuebo Liu

**Affiliations:** ^1^Department of Cardiology, Tongji Hospital, School of Medicine, Tongji University, 200065 Shanghai, China

**Keywords:** acute coronary syndromes, plaque rupture, plaque erosion, calcified nodules, atherosclerosis

## Abstract

Acute coronary syndrome (ACS) is the most severe form of ischemic heart disease. Although it is caused by atherosclerotic plaque thrombosis or nonatherosclerotic causes, its pathophysiological mechanism of ACS is not fully understood, and its concept is constantly updated and developed. At present, the main pathophysiological mechanisms include plaque rupture, plaque erosion, calcified nodules (CN) and non-atherosclerotic causes such as coronary vasospasm and myocardial bridging (MB). These mechanisms may overlap and coexist in some ACS patients. Therefore, the pathophysiological mechanism of ACS is complex, and is of great significance for the diagnosis and treatment of ACS. This review will discuss the pathophysiological mechanisms of ACS to provide new thoughts on the pathogenesis, diagnosis and treatment of ACS.

## 1. Introduction

Ischemic heart disease remains a major source of morbidity and mortality worldwide, with acute coronary syndromes (ACS) being the most critical. ACS is characterized by a sudden decrease in blood supply to the heart and is often caused by thromboembolic coronary or non-atherosclerotic etiologies, resulting in ST-segment elevation myocardial infarction (STEMI), non-STEMI (NSTEMI), and unstable angina (UA) [1]. Each year, more than 7 million people worldwide are estimated to be diagnosed with ACS [2]. Therefore, the pathophysiology of ACS has been extensively studied, and three main pathophysiology mechanisms have been proposed. The first is plaque rupture, mainly involving atherosclerotic plaques rich in lipids and thin fibrous caps. Metalloproteinases (MMP) degrade the fibrous cap resulting in rupture and necrosis of the core exposed to the vascular lumen, leading to platelet activation and thrombosis [3]. In a small proportion of cases, plaque rupture occurs at the site of calcified nodules (CN). Plaque rupture may occur with or without systemic inflammation [4]. The second is plaque erosion, where thrombus formation occurs mainly in the area of endothelial desquamation adjacent to the atherosclerotic plaque, without destroying the fibrous cap covering the plaque tissue [5]. The third mechanism is caused by non-atherosclerotic causes in the absence of obvious thrombosis, such as coronary vasospasm and myocardial bridging (MB) [6]. These multiple pathophysiological mechanisms may coexist in some patients with ACS. The concept of ACS is also developing. For example, plaque erosion gradually dominates in the era of intensive lipid lowering, and the concept of the “vulnerable patient” also leads to new in the management of ACS patients. This review will focus on the research progress on the pathophysiology of ACS, aiming to provide new insights in the pathogenesis and treatment of ACS.

## 2. Plaque Rupture

Plaque rupture occurs when the fibrous cap covering the lipid-rich necrotic core breaks or fissures [7], allowing blood containing potential clotting proteins to come into contact with procoagulant substances (such as tissue factors) in the lipid core, which will trigger thrombosis [8]. Plaque rupture is firstly found in autopsy, and is typically characterized by a large lipid core filled with macrophage foam cells and a variety of debris, and a thin fibrous cap (<65 mm) rich in extracellular matrix (ECM) covering the lipid necrotic core of the plaque [9]. These types of plaques which are known as thin-capped fibroatheroma (TCFA), also known as the “vulnerable plaque”. In the past decades, the concept of the “vulnerable plaque” has been dominant in the pathogenesis, diagnosis and treatment of ACS, and autopsy studies consistently found that plaques of this morphology were responsible for the majority of fatal myocardial infarcts. A large number of studies indicated that rupture of TCFA is related to the increased breakdown and decreased synthesis of interstitial collagen that forms the fibrous cap [10]. Interstitial collagen produced by vascular smooth muscle cells (VSMCs) provides strength to the fibrous cap of the plaque. There are fewer VSMCs in the TCFA plaque area, resulting in an impaired ability to repair the fibrous cap that protects the plaque [11, 12]. When stimulated by inflammation and other injuries, the macrophages in plaques increased expression of MMP and other proteases that can degrade interstitial collagen [13]. In TCFA, MMP-1, 8, and 13 attack fibrillary collagen types I and III, which are produced primarily by VSMCs to protect plaques from rupture, resulting in breakdown of the fibrous cap.

Coronary thrombosis caused by plaque rupture can be classified as with or without systemic inflammation. Because of the increasing attention paid to direct anti-inflammatory interventions targeting atherosclerosis (AS) in recent years, this classification of plaque rupture may have more significance. The first concept is plaque rupture with systemic inflammation. ACS patients often have a marked increase in C-reactive protein (CRP), which provides evidence of systemic inflammation in ACS [14]. Inflammatory mechanisms are widely recognized as key regulators of fibrous cap fragility and lipid core thrombosis [15]. When macrophages are activated, they produce enzymes that degrade plaque matrix components, such as MMP and cathepsin [16]. Therefore, increase the amounts of activated proteases can enhance the breakdown of plaque ECM. Adaptive immunity is also involved in coronary plaque instability. The number of proinflammatory CD34+ T cells is increased in ACS patients, while the number of T helper cell 17 (Th17) and CD4+CD25+ regulatory T cells (Tregs) are decreased [4]. Activated Th17 can promote the formation of thick collagen, which may further increase plaque stability, while plaques tend to be unstable when Th17 is reduced. Tregs maintain immune homeostasis through immunosuppression, by releasing anti-inflammatory factors such as interleukin (IL)-10 and transforming growth factor-β1 (TGF-β1). Compared to patients with stable angina and healthy controls, the amount of circulating Tregs is reduced in patients with ACS [17], resulting in insufficient immunosuppression and severe inflammatory reactions. Therefore, when plaque rupture is accompanied by systemic inflammation, anti-inflammatory treatments such as colchicine [18], methotrexate, and IL-1β neutralizing antibodies [19] may be of benefit to ACS patients [20]. Another etiology of ACS is plaque rupture without systemic inflammation. In plaque rupture without systemic inflammatory activation, other mechanisms may be involved in the process of plaque rupture, including extreme mood disorders, intense physical exertion, and abnormal mechanical stress in the arterial wall. Plaque rupture caused by psychological stress may be related to sympathetic nervous system activation and catecholamine release, as it increases heart rate, blood pressure, and coronary artery constriction, which favors plaque rupture and platelet activation [21, 22]. Although physical or mental stress may not by itself cause coronary thrombosis, it may lead to destabilization of plaques that are already predisposed to trigger events.

The “Vulnerable plaque” was initially studied at autopsy. Therefore, these studies have a limited number of patients with TCFA morphology who did not rupture or even triggered ACS resulting in death. The development of several emerging technologies, including endovascular imaging, has added new insight in the role of TCFA in the pathophysiology of ACS. The PROSPECT (Providing Regional Observations to Study Predictors of Events in the Coronary Tree) study, which used intravascular ultrasound (IVUS) to assess plaque status in 3.4 years of TCFA follow-up, showed that less than 5% of TCFA resulted in clinical events [23]. In addition, a recent study of 5869 cases of sudden cardiac death confirmed by autopsy found that only less 24% had evidence of acute plaque rupture, while 97% of sudden cardiac death patients had myocardial hypertrophy and/or myocardial fibrosis [24]. This suggests that the interplay between preexisting cardiac hypertrophy, fibrosis, and acute ischemia may play a more important role in the pathogenesis of sudden cardiac death than plaque rupture alone. Therefore, the “vulnerable plaque” is considered to be a misnomer, and most TCFA appear to be stable and may not trigger clinical events [23]. Furthermore, TCFA plaques may acquire more stable features. Moreover, the occurrence of plaque rupture usually occurs in asymptomatic ACS patients, which makes it difficult to predict [25, 26]. In the era of great emphasis on the control of atherosclerotic risk factors, cardiovascular experts proposed to focus on the “vulnerable patient” rather than the “vulnerable plaque”, indicating that ACS is a systemic disease [27]. The “Vulnerable patient” is defined as those who are susceptible to ACS or sudden cardiac death. These patients have three characteristics: vulnerable plaque, vulnerable blood, and vulnerable myocardium [28]. The concept of vulnerable plaque has been described previously in this review. Vulnerable blood refers to blood that is in a hypercoagulable state and prone to thrombosis caused by the imbalance between coagulation, anticoagulation and fibrinolysis in the body. Vulnerable myocardium refers to myocardium that is prone to fatal arrhythmias due to electrical instability of cardiomyocytes. The concept shift from the simply “vulnerable plaque” to the “vulnerable patient” has been widely accepted by cardiologists. The management of coronary heart disease should not be regarded as a disease, but as a spectrum of disease. Therefore, the concept of focusing on a single “vulnerable plaque” is no longer appropriate. In order to reduce the risk of coronary heart disease, full implementation of comprehensive interventions, such as the widespread use of lipid-lowering therapies, antiplatelet therapy, improved blood pressure and diabetes control, smoking cessation, and better diet and lifestyle, is more important [28, 29].

Under the background of effective and intensive control of risk factors such as hyperlipidemia and hypertension, the pathophysiology of human AS has also changed. Lipid-lowering reduces the lipid core, reduces lesion size, plaque lipid accumulation and inflammatory cell reduction, and even promotes plaque regression and healing [26]. Lipid-lowering is associated with an increase in the proportion of fibrous tissue such as ECM in the plaque, thereby enhancing the stability of the fibrous cap [3]. Thus, plaque rupture may be decreasing as therapeutics improve. However, with the use of statins and other drugs with significant low-density lipoprotein (LDL) lowering effects, ACS events still frequently occur. This suggests that mechanisms that are less responsive to the control of risk factors may be important for ACS today, and has spurred interest in mechanisms other than plaque rupture that may trigger ACS.

## 3. Plaque Erosion

Reconsideration of the concept of the “vulnerable plaque” has led to interest in an alternative mechanism of ACS, namely plaque erosion. This disruption has long been observed by pathologists, and recent studies highlight the growing importance of plaque erosion as a mechanism for ACS. The lesions of coronary events caused by erosion are in some ways diametrically opposed to the morphological features of TCFA. Eroded plaques have intact fibrous caps and high concentrations of ECM molecules. For example, the content of proteoglycan and glycosaminoglycans increases, especially hyaluronic acid [5]. CD44, the cell surface receptor of hyaluronic acid, is significantly localized in eroded plaques [30]. VSMCs are abundant in eroded plaques, leukocytes such as macrophages are less aggregated, and lipids are lacking [31]. Different from the “red thrombus” rich in fibrin and red blood cells in plaque rupture, the thrombus in plaque erosion is the “white thrombus” rich in platelets [4]. This suggests that effective antiplatelet therapy without stents may be effective against ACS caused by plaque erosion, thereby avoiding stent-related complications [32, 33]. The EROSION study (Effective Anti-Thrombotic Therapy Without Stenting: Intravascular Optical Coherence Tomography-Based Management in Plaque Erosion) demonstrated the feasibility and safety of antithrombotic therapy instead of stent placement in ACS patients caused by plaque erosion, and provided a new option for the treatment of patients with plaque erosion [34]. Now that there is more effective therapy for the traditional risk factors of AS, plaque erosion may have greater clinical significance. Plaque erosion causes about 1/3 of ACS, and most of them are NSTEMI. In recent decades, the clinical presentation of ACS has shifted and NSTEMI has surpassed STEMI, which may be related to the increasing proportion of patients with plaque erosion.

Plaque erosion also differs from plaque rupture in terms of epidemiology and clinical manifestations. Plaque erosion is more common in women, younger patients, and those with a lower prevalence of traditional cardiovascular risk factors [35, 36]. Patients with erosion may have a nonocclusive thrombus or an occlusive thrombus that easily embolizes distally because of less disruption of arterial integrity and a larger lumen [37]. Compared with patients with plaque rupture, ACS patients with plaque erosion have lower plaque burden, less complex lesions, and fewer adverse cardiac events. Patients with plaque erosion have low levels of inflammatory markers, such as CRP and low leukocyte levels, and plaque erosion has a more favorable lipid profile compared with those with plaque rupture [38, 39, 40]. Plaque erosion affects the left anterior descending coronary artery more frequently than the right or circumflex coronary arteries [41]. Patients with plaque erosion had higher levels of hemoglobin concentration than those with plaque rupture, which may be related to the fact that blood concentration increases blood viscosity, resulting in high endothelial shear stress, and thus activation of platelets and the coagulation system [40].

Plaque erosion usually occurs in lesions lacking local intimal endothelium. The death and desquamation of intimal endothelial cells (ECs) plays an important role in plaque erosion thrombosis. Optical coherence tomography (OCT) can provide the submicroscopic structure of the inner membrane surface, which can provide the “optical biopsy” to evaluate the plaque status in more detail. The typical feature of plaque rupture under OCT is the discontinuity of the plaque fibrous cap [42]. An ACS and mural thrombus without a discernable plaque fissure is a definite diagnosis of plaque erosion by OCT, which is a diagnosis of exclusion [42]. Patients with ACS do not have a diagnosis of ruptured fibrous caps may instead have plaque erosion, especially if accompanied by thrombosis or irregular intimal surfaces [42]. In addition to measurements that reflect coronary structure, such as OCT, some invasive measures reflecting coronary function can be useful, such as coronary index of microcirculation resistance (IMR) and coronary flow reserve (CFR). Coronary microcirculation is a complex vascular network with vessels ≤500 μm in diameter and coronary microvascular dysfunction (CMD) can affect IMR and CFR results. Coronary microcirculation has an important role in the regulation of blood flow and oxygen supply and nutrient exchange in the myocardium. When CMD occurs, it can play an important pathological role in ACS after reperfusion therapy or in absence of severe epicardial stenosis. The identification of CMD is of great significance for the diagnosis, treatment and prognosis of ACS.

In recent years, studies on the pathophysiology of plaque erosion, innate immunity and ECs death, toll-like receptor-2 (TLR2) upregulation, formation of neutrophil extracellular traps (NETs), and endothelial-to-mesenchymal transition (EndMT) have discovered relatively new mechanisms involved in the pathogenesis of plaque erosion, which will be explained in detail below.

### 3.1 Innate Immunity and Endothelial Cells Death

The decreased content of macrophages in eroded plaques suggests that inflammation may play a minor role in plaque erosion compared to plaque rupture. However, there are a number of unique innate immune responses that are involved in plaque erosion. Both the mechanisms of desquamation of the basal surface of ECs and the underlying basement membrane and ECs death may increase the risk of endothelial desquamation, thereby promoting plaque erosion. The adhesion of ECs to the underlying membrane relies primarily on the nonfibrous archetypes type IV collagen and laminin [43]. Therefore, degradation of collagen IV by collagenase type IV such as MMP-2 can lead to desquamation of ECs and subsequent plaque erosion [44]. Studies have shown that proinflammatory cytokines can induce the expression of MMP-14, which is a precursor of MMP-2 activator and helps to process inactive zymogen into mature MMP-2, thereby degrading type Ⅳ collagen of the basement membrane. This suggests that MMP-14-mediated activation of MMP-2 is involved in the mechanism of plaque erosion, while MMP-1,8 and 13, which play an important role in plaque rupture, may not play a significant role in plaque erosion [5]. ECs death, especially apoptosis, disrupts the continuity of the endothelial monolayer. Some studies have found that apoptosis occurs when ECs are exposed to dangerous stimuli such as hypochlorous acid, which may be another mechanism for desquamate of ECs in eroded areas [45, 46]. Hypochlorous acid is a potent oxidant produced by the processing of myeloperoxidase (MPO), which is often expressed in inflammatory cells such as macrophages in the subcutaneous region of plaques [47]. It has been found that thrombi covering eroded plaques have a higher concentration of MPO positive cells than observed in ruptured plaques, and MPO may contribute to plaque erosion by promoting ECs apoptosis by disrupting intimal integrity [48]. In addition, local flow disturbances and abnormal flow shear forces are potential causes of ECs death and endothelial monolayer damage [48].

Partial adaptive immunity may also contribute to plaque erosion. Studies have shown that CD8+ T lymphocytes are more often present in eroded plaques than in ruptured plaques [5]. The local concentrations of granzyme B and perforin are increased at the site of ACS lesions with plaque erosion. Therefore, CD8+ T lymphocyte-derived mediators may cause ECs damage and promote plaque erosion.

### 3.2 Toll-Like Receptor-2 Upregulation 

TLR2 is an important innate immune recognition receptor, which is significantly upregulated at plaque sites and circulation in patients with plaque erosion [31, 49]. Studies have shown that TLR2 activation promoted ECs injury and may lead to desquamation of ECs in areas of plaque erosion. When cultured human ECs were exposed to TLR2 agonists, the expression of leukocyte adhesion molecules, such as intercellular adhesion molecule-1 (ICAM-1) or E-selectin, was increased [50, 51, 52]. TLR2 activation also promotes the expression of IL-8, which acts as a chemoattractant for granulocytes [53]. Thus, TLR2 activation in ECs results in desquamation and neutrophil recruitment. When ECs desquamate, neighboring cells rapidly migrate and restore intimal integrity. It has been found that this repair is delayed when human ECs are exposed to TLR2 agonists *in vitro * [5]. This suggests that TLR2 stimulation in ECs contribute to plaque erosion. This has led to a search for endogenous TLR2 ligands associated with plaque erosion, which, as previously described, accumulates in large amounts of proteoglycans and hyaluronan. Previous studies have shown that hyaluronan fragments can activate TLR2 as danger signals, suggesting that hyaluronan may be an endogenous factor in erosion-related endothelial dysfunction [49]. The expression of E-selectin, vascular cell adhesion molecule-1 (VCAM-1) and IL-8 was increased in cultured ECs stimulated by hyaluronic acid, and the activation of caspase-3 was increased [3, 51, 54]. This suggests that TLR2 is also involved in promoting the sensitivity of ECs to apoptotic stimuli. Hyaluronic acid, a major component that erodes the ECM of diseased cells, can act as an endogenous ligand of TLR2 to promote ECs apoptosis. In addition to the hyaluronic acid fragment, other endogenous danger associated molecular patterns (DAMPs) or pathogen associated molecular patterns (PAMPs) can act on TLR2 or other innate immune receptors, thus promoting endothelial activation associated with plaque erosion. Such stimuli may include oxidized lipids, fatty acids and microbial composition [3].

### 3.3 Formation of Neutrophil Extracellular Traps

It is generally believed that the pathogenesis of plaque erosion is related to a 2-hit process, which means that the thrombosis caused by plaque erosion can be divided into two stages [48]. Low levels of innate immune activation of ECs in the vascular lumen are considered to be the first hit. Perturbation of local blood flow around the coronary plaque may contribute to ECs activation. Perturbation of blood flow may activate innate immune TLR2, cause the death and detachment of luminal ECs, and impair the integrity of the endothelial monolayer [48]. In addition, the ability of adjacent cells to recover exposed subintima may be impaired. After shedding of the inner membrane, the exposed basement membrane attracts platelets, which can be activated by contact with components of the arterial ECM, such as collagen [55]. In the second hit, activated ECs produce chemokines such as IL-8 to recruit leukocytes [56]. Moreover, the release of granule contents by activated platelets induces a series of reactions in polymorphonuclear leukocytes [57]. Specifically, the activation of granulocytes on the inner membrane surface can accentuate the damage through reactive oxygen species (ROS), proteases and the formation of NETs [58]. NETs are derived from neutrophils that undergo NETosis, a specific type of cell death. Neutrophils, which die of NETosis, release unwound strands of DNA that are decorated with a variety of proteins, including MPO and a series of serine proteases [58]. NETs are rich in MPO, which can be used as a reactor to produce large amounts of hypochlorous acid. NETs can also obtain tissue factors and inflammatory factors, such as IL-1α, from blood. NETs can also trap platelets and fibrin to activate thrombin, leading to local white thrombosis [48]. Thus, NETs constitute a solid-state reactor, which results in proinflammatory and prothrombotic cascades of acute thrombosis and leads to the ACS. NETs also enhance endothelial stress and stimulate apoptosis and desquamation of endothelial monolayers, demonstrating that NETs can contribute to pathological processes associated with plaque erosion [59]. Studies have shown that NETs in erosive plaques are more abundant than those in rupture-related plaques in human atherosclerotic plaques, indicating that NETs play a central role in plaque erosion. Some studies showed that statins may actually increase neutrophil formation of NETs [60]. Statin therapy undoubtedly reduces the overall risk of ACS and is one of the most important tools for anti-AS drug therapy. However, there is the possibility that these drugs may increase the risk of thrombosis due to plaque erosion by promoting NETosis. This may also be one of the reasons why plaque erosion is less responsive to lipid-lowering than plaque rupture.

The generation of NETs relies on peptidyl arginine deiminase-4 (PAD-4), an enzyme that converts arginine to citlinline, thus changing the electrical properties of amino acids in histones [58]. In this manner, PAD-4 disrupts ionic interactions between DNA and histones that are wound around the DNA strand, which results in the DNA strands not being wound. Because NETs play a more important role in plaque erosion, interventions targeting NETs may be more effective in ACS erosion than those caused by rupture. Deoxyribonuclease and PAD-4 inhibitor therapy are worthy of consideration. Deoxyribonuclease can degrade the DNA strands that form the skeleton of NETs and reduce the formation NETs.

### 3.4 Endothelial-to-Mesenchymal Transition

Mature ECs can exhibit considerable heterogeneity and can transdifferentiate into mesenchymal like cells when they are stimulated by a variety of factors, a biological process called EndMT [61]. EndMT is characterized by activation of EndMT markers such as mesenchymal markers, VSMCs markers, ECM and pro-inflammatory proteins, and enhanced proliferation and migration, leading to major changes in ECs morphology, polarity, and function [62]. EndMT is a major aspect of endothelial dysfunction and may play an important role in promoting plaque erosion. When ECs are stimulated by oxidative stress, hypoxia, low shear stress and inflammation, ECs undergo EndMT through TGF-β and other pathways [61]. In turn, EndMT participates in the disruption of VE-cadherin and disassembly of adherens junctions, which ultimately leads to the impairment of endothelial integrity and promotes thrombosis [3]. However, the specific mechanism of EndMT in plaque erosion and ACS needs to be further studied, because there is no consensus on whether it is “harmful” or “beneficial” in ACS.

## 4. Calcific Nodule 

Calcified plaques are divided into superficial calcific sheets, the most common type, followed by eruptive CN and calcified protrusion [63]. This review is mainly focused on CN. CN is one of the least common compared with plaque rupture and plaque erosion but can still lead to acute coronary thrombosis in ACS patients. It is defined as crater-like and prominent nodular calcifications with luminal surface attached thrombus, and is another potential cause of ACS. CN has a unique plaque morphology, characterized by nodular calcification leading to disruption of the fibrous cap and covering the lumen thrombus [64]. Destruction of the fibrous cap and thrombosis are caused by fragmentation of the necrotic core calcifications, which are surrounded by hard collagen-rich calcifications in the coronary arteries that are susceptible to mechanical stress [64]. CN occurs mainly in the proximal to midsection of the highly curved right coronary artery, where the range of motion of the coronary hinge is greatest throughout the cardiac cycle [64, 65]. Under the combined action of the surrounding rigid collagen calcification and external mechanical stress, the necrotic core calcification breaks into a large number of fragments, which damage the capillaries and cause intraplaque hemorrhage. The plaque rapidly increases in size and protrudes into the lumen, destroying the fibrous cap and endothelium, and forming CN [64]. OCT is the gold standard for the diagnosis of CN, which are characterized by clumps of CN protruding into the lumen on the surface of lamellar calcified plaques, accompanied by fibrous cap rupture and thrombosis. This should be differentiated from red thrombus. Patients with CN may have worse cardiac outcomes such as target lesion failure and recurrent ACS after percutaneous coronary intervention (PCI) because of an increased risk of coronary calcification related cardiac events. One study showed that 82.4% of target lesion failures after stent implantation were caused by CN [66]. Proper plaque modification is the key to the treatment of CN. Common plaque modification techniques include coronary rotational atherectomy (CRA), excimer laser coronary angioplasty (ELCA) and coronary intravascular lithotripsy (IVL).

## 5. Non-Atherosclerotic Causes

In addition to plaque rupture, plaque erosion, and CN, there are some non-atherosclerotic causes of ACS, leading to acute myocardial ischemia. Common non-atherosclerotic causes of ACS include coronary vasospasm, spontaneous coronary artery dissection (SCAD), MB, stress-induced cardiomyopathy (Takotsubo syndrome) and coronary artery embolism due to thrombus from elsewhere in the body causing obstruction.

### 5.1 Coronary Vasospasm 

Coronary vasospasm is defined as transient epicardial coronary artery constriction that leads to vascular occlusion and even myocardial ischemia. Cardiologists generally agree that we should pay attention to the prevention and treatment of type 2 myocardial infarction and the existence of myocardial infarction with non-obstructive coronaries (MINOCA). An important cause of type 2 myocardial infarction and MINOCA is coronary vasospasm. Coronary vasospasm is more common in men, with an age ranging between 40 and 70 years. It is more common in the Japanese population. The exact pathophysiological mechanism of coronary vasospasm is still unclear and may be the result of a variety of factors, including autonomic nervous system disorders, endothelial dysfunction, inflammation, oxidative stress, VSMCs hyperresponsiveness and genetics [67, 68, 69]. Coronary vasospasm and the sympathetic and parasympathetic nervous systems of the autonomic nervous system are both related, which reflects the complexity of their relationship. Coronary vasospasm occurs predominantly in the early morning. Circadian changes and acetylcholine can cause coronary spasm. This supports the role of the parasympathetic nervous system in the pathophysiology of coronary vasospasm [6]. The increase in catecholamine and adrenergic receptor activity and the decrease in parasympathetic activity after an ischemic attack suggest that coronary vasospasm occurring at this time may be related to the involvement of the sympathetic nervous system. Vascular physiological dysfunction also plays an important role in the pathogenesis of coronary vasospasm. Endothelial nitric oxide (NO) release is decreased in patients with coronary vasospasm [67], which causes dysregulation of endothelial relaxation and predisposes to coronary vasospasm. There are also drugs that can induce coronary vasospasm. These drugs include cocaine, amphetamines, marijuana, and alcohol [6]. The most reliable diagnostic method for coronary vasospasm is the ergonovine test, in which intramuscular or intracoronary injection of ergonovine is performed at the time of coronary angiography to provoke coronary vasospasm. This is manifested by a reduction in the coronary vessel diameter to a certain threshold or an arbitrary reduction in the vessel diameter accompanied by chest pain or ischemic electrocardiographic changes. At present, cardiac magnetic resonance (CMR) has become the gold standard for the diagnosis of MINOCA, and late gadolinium enhancement (LGE) can locate the area of myocardial injury. However, the use of CMR in the diagnosis of coronary vasospasm is still controversial, and myocardial blood flow reduction or myocardial perfusion dyssynchrony can only indicate the possibility of coronary vasospasm. Nitrates, calcium channel blockers (CCB) and smoking cessation are effective ways to relieve and treat coronary vasospasm.

### 5.2 Spontaneous Coronary Artery Dissection

SCAD is a cause of ACS, especially in younger patients without risk factors for AS, and is common in women [70]. SCAD is a rare coronary artery disease with unclear etiology. It has been found to be associated with factors such as muscle fiber dysplasia, hormonal body changes, childbirth, and connective tissue disease. SCAD is secondary to the formation of a false lumen in the coronary intima-media, which often leads to intramural hematoma formation and compression of the vascular lumen, resulting in coronary blood flow obstruction and even ACS [71]. The triggering mechanism for SCAD is not well understood. There are two main mechanistic theories. The first suggests a primary rupture or dissection of the inner membrane, followed by blood infiltration and accumulation from the arterial lumen into the wall, forming a false lumen leading to compression and coronary artery stenosis [72]. The second mechanism of the formation of an inter-membranous hematoma is the rupture and hemorrhage of the vessels that nourish the wall of the vessel. The hemorrhage may fill the false lumen and form a closed space. The vascular lumen is compressed with this “outside-in” style resulting in ischemia [72]. It is unclear whether endothelial dysfunction and vasospasm are associated with SCAD. Patients with suspected SCAD as the cause of ACS should undergo coronary angiography to confirm the diagnosis. If the diagnosis cannot be confirmed by coronary angiography or during PCI treatment, OCT can be used to make the diagnosis. OCT images of SCAD are as follows: a separation of the intima and media from the adventitia, with or without communication with the vessel lumen (intimal tear) [42]. 80% of SCAD patients can be cured by medical treatment, such as anticoagulation, antiplatelet agents, β-blockers, statins, and anti-angina medication. In clinically stable patients, medical therapy is preferred over immediate revascularization and should be considered if high-risk features are present.

### 5.3 Myocardial Bridging 

MB is a congenital malformation of the coronary artery in which a segment of the coronary artery that would otherwise travel on the epicardium penetrates the muscular layer of the heart [73]. This segment of the coronary artery is called the mural coronary artery, and the cardiac muscle covering it is called the MB. MB as a congenital anomaly most commonly involving the left anterior descending artery. Most MB are asymptomatic, but their hemodynamic effects are associated with the thickness and length of the bridge, and MB has been associated with stable angina, ACS and sudden cardiac death [74]. According to the depth of the mural coronary artery of the MB, MB is divided into a superficial type running in the ventricular groove and a deep type running close to the right ventricular septum. In the cardiac cycle, the myocardial fibers in the mural coronary artery segment twist and contract, compressing the blood vessels, resulting in endothelial injury and a decrease in coronary flow reserve, which may further cause luminal stenosis and atherosclerotic changes in the vessel wall. In severe cases, myocardial ischemia and even acute myocardial infarction may occur [75]. Coronary CT angiography can directly observe the relationship between the coronary artery and the myocardium, and diagnose the MB by showing the coronary vascular segment running in the myocardium. The appearance of MB on coronary CT angiography is as follows: the mural coronary artery traveled in the myocardium for a certain distance and then is exposed on the surface of the myocardium. The mural coronary artery is slightly thinner than the adjacent normal vessels at both ends, with slightly blurred edges. The MB generally has a favorable prognosis. Symptomatic patients should be given appropriate treatment, such as β-blockers and CCB. When drug treatment is ineffective, surgical treatment can be considered.

### 5.4 Stress-Induced Cardiomyopathy

Stress-induced cardiomyopathy is a transient, reversible disease that occurs after a stressful event and is characterized by transient abnormalities in left ventricular wall motion, with clinical manifestations similar to ACS, especially STEMI on electrocardiogram (ECG), and occurs mostly in postmenopausal women [76]. It is also called Takotsubo syndrome (TTS) because the shape of the left ventricle in this lesion is similar to the octopus pot in Japan, which was first discovered by Japanese doctors around 1990. The exact pathophysiological mechanisms of TTS are still unclear. Sympathetic stimulation leading to increased circulating and local cardiac tissue catecholamine levels is thought to be the major pathology [76, 77]. Elevated catecholamines can induce vascular spasm or cause direct myocardial toxicity, which causes TTS, together with increased cardiac load, resulting in an acute supply-demand mismatch and even post-ischemic shock [78]. Other pathophysiological mechanisms leading to TTS include psychological stress (depression, anxiety), coronary artery or microvascular spasm, metabolic and energy changes, and inflammation [76, 78]. The treatment of TTS is based on removing predisposing factors to avoid stress factors, actively treating the primary disease, and symptomatic and supportive treatment.

### 5.5 Coronary Artery Embolism 

Coronary embolism refers to a pathological process in which emboli from the heart or proximal artery wall or other parts of the body enter the coronary artery through the blood, blocking the coronary blood flow and leading to myocardial ischemia, causing myocardial injury or even necrosis. It may be responsible for 3–5% of ACS. There are multiple sources of embolism, such as detachment of blood thrombus at other sites, tumors, fat, air, and foreign material [79]. Infective endocarditis and valve replacement are considered to be the most common causes of coronary embolism. The embolism obstructs or occludes coronary blood flow, ultimately leading to myocardial ischemia and subsequent ACS. Coronary artery embolism is divided into three types: direct, paradoxical and iatrogenic [6]. Direct coronary embolism refers to an embolism caused by material from left cardiac structures, including the atrium, ventricle, valve, and left myxomas, and results in severe obstruction of the coronary artery. Both atrial fibrillation (AF) and valvular heart diseases such as rheumatic valvular heart disease combined with AF can cause coronary artery embolism, and should be paid special attention to in the current era of the increasing incidence of AF. Paradoxical coronary embolism is caused by the detachment of thrombus that forms outside the coronary artery, such as deep vein thrombosis, and travel in the blood into the coronary arteries to cause ACS [80]. Last but not least, iatrogenic coronary embolism occurs during interventions in the catheterization lab or operating room, secondary to entry of surgical material, air or thrombus into the coronary arteries [6]. Air embolism is the most common iatrogenic coronary embolism, which occurs more commonly during a PCI. The most common coronary artery involved is the right coronary artery, since the right coronary artery orifice is higher than the left coronary artery when the patient is lying flat during the PCI. The diagnosis of coronary embolism needs to be based on the patients’ history, coronary angiography, and other imaging studies. The treatment options for a coronary embolism are based on their etiology. Common treatment options for coronary embolism include thrombus aspiration, stent implantation, balloon angioplasty, drug anticoagulation, and anti-infective drug therapy.

## 6. Conclusions and Discussion

**Fig. 1.**
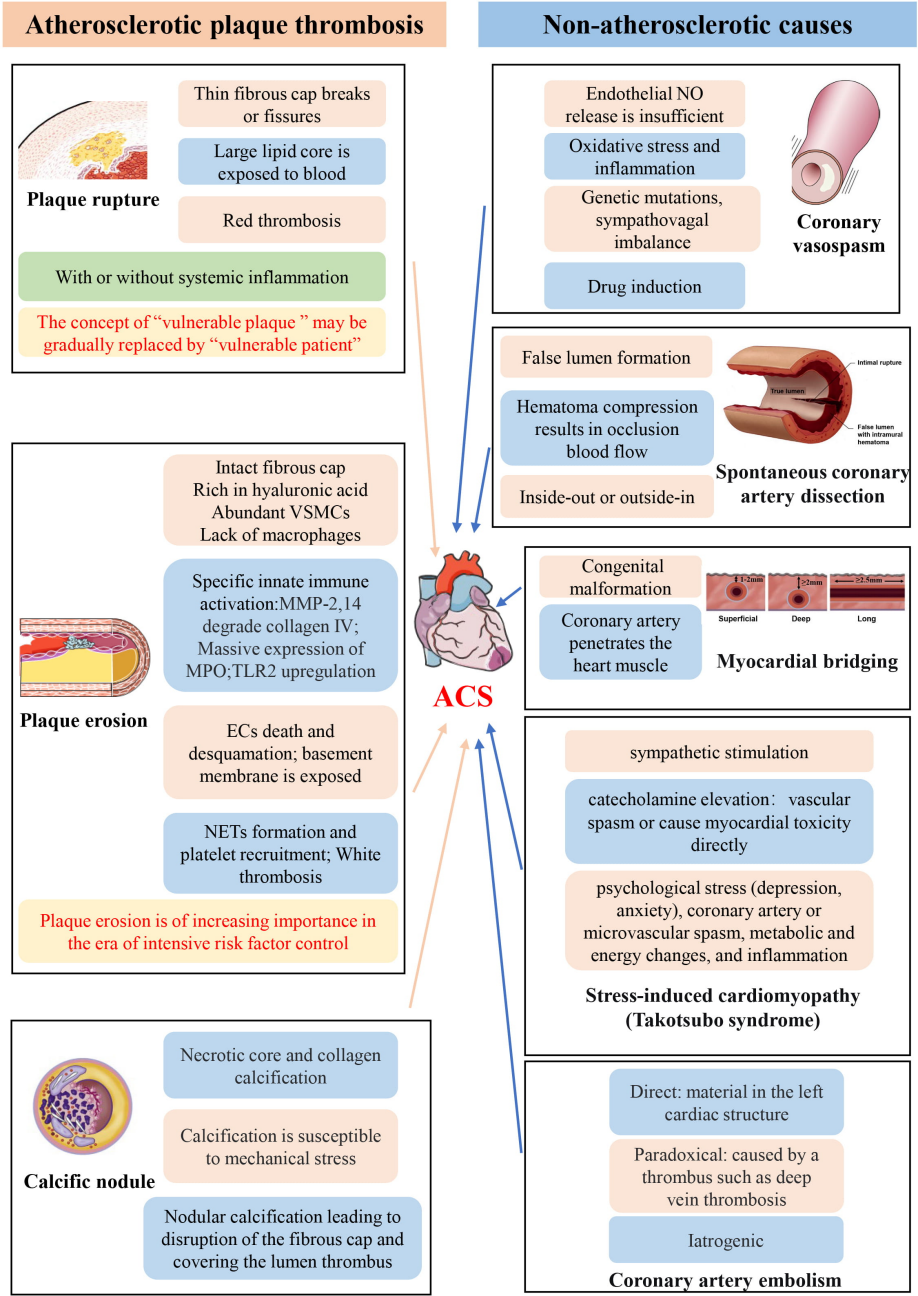
**Schematic representation of the pathophysiological mechanism of ACS**. It is noted that the illustrations of calcified nodules [64], coronary vasospasm [4], spontaneous coronary dissection [81] and myocardial bridging [73] are from the article of other researchers. The full names of all abbreviations in the figure are given in the “Abbreviations” section.

In summary, some ACS are due to thrombosis caused by atherosclerotic plaques, and others are due to non-atherosclerotic causes. Several pathophysiological mechanisms of ACS described in this paper are shown in Fig. 1. (Ref. [4, 64, 73, 81]). The causes of thrombosis in ACS include plaque rupture, plaque erosion and CN. Their pathophysiological mechanisms are quite different, and it is particularly important to distinguish them and carry out targeted treatment. However, at present, they are mainly distinguished by intravascular imaging technology, which is an invasive procedure with certain risks. Therefore, there is a need to develop and validate biomarkers that can distinguish plaque erosion from rupture and further guide treatment without the need for invasive technologies such as IVUS and OCT, and some coronary function indicators such as IMR and CFR. This is of great significance for the development of precision medicine for ACS. Recent studies have found that plasma Trimethylamine N-Oxide (TMAO) is independently associated with plaque rupture in patients with STEMI, and may be a useful biomarker for plaque rupture, especially in differentiating plaque rupture from plaque erosion [82]. In addition, some non-coding RNA such as MicroRNA (miRNA) also have specific expression patterns in plaque rupture. Therefore, non-coding RNA will have an important role in the identification of plaque rupture and erosion [83]. NETs-related components and IL-8 have been highly correlated with plaque erosion. In the future, specific biomarkers, drug interventions, and high-resolution non-invasive imaging will all be investigated. Two new concepts deserve special attention. First, plaque erosion is gradually becoming the most important mechanism of ACS, and second, the concept of the “vulnerable patient” is gradually replacing the concept of the “vulnerable plaque”. In addition, there are some non-atherosclerotic causes of ACS that are also worthy of attention. Although they account for a relatively small proportion of ACS, they still can result in major adverse events. The pathogenesis of ACS caused by non-AS is unique, and its identification with traditional plaque rupture and erosion is of great significance for the treatment of ACS caused by non-AS. In conclusion, the pathophysiological mechanism of ACS is complex and diverse and will require further investigation to determine new therapeutic options.

## Abbreviations

ACS, acute coronary syndromes; STEMI, ST-segment elevation myocardial infarction; NSTEMI, non-ST-segment elevation myocardial infarction; UA, unstable angina; MMP, metalloproteinases; CN, calcified nodules; ECM, extracellular matrix; TCFA, thin-capped fibroatheroma; VSMCs, smooth muscle cells; AS, atherosclerosis; CRP, C-reactive protein; IL, interleukin; Th17, T helper cell 17; Tregs, regulatory T cells; TGF-β1, transforming growth factor-β1; IVUS, intravascular ultrasound; LDL, low-density lipoprotein; ECs, endothelial cells; OCT, optical coherence tomography; IMR, index of microcirculation resistance; CFR, coronary flow reserve; CMD, coronary microvascular dysfunction; QFR, quantitative flow ratio; TLR2, Toll-like receptor-2; NETs, neutrophil extracellular traps; EndMT, endothelial-to-mesenchymal transition; MPO, myeloperoxidase; ICAM-1, intercellular adhesion molecule-1; VCAM-1, vascular cell adhesion molecule-1; DAMPs, danger associated molecular patterns; PAMPs, pathogen-associated molecular patterns; ROS, reactive oxygen species; PAD-4, peptidyl arginine deiminase-4; PCI, percutaneous coronary intervention; CRA, coronary rotational atherectomy; ELCA, excimer laser coronary angioplasty; IVL, coronary intravascular lithotripsy; SCAD, spontaneous coronary artery dissection; MB, myocardial bridge; MINOCA, myocardial infarction with non-obstructive coronaries; NO, nitric oxide; CMR, cardiac magnetic resonance; LGE, late gadolinium enhancement; CCB, calcium channel blockers; ECG, electrocardiogram; AF, atrial fibrillation; TTS, Takotsubo syndrome; TMAO, Trimethylamine N-Oxide; miRNA, MicroRNA.
